# Global transcriptome analysis reveals potential genes associated with genic male sterility of rapeseed (*Brassica napus* L.)

**DOI:** 10.3389/fpls.2022.1004781

**Published:** 2022-10-21

**Authors:** Jianxia Jiang, Pengfei Xu, Junying Zhang, Yanli Li, Xirong Zhou, Meiyan Jiang, Jifeng Zhu, Weirong Wang, Liyong Yang

**Affiliations:** ^1^Crop Breeding and Cultivation Research Institute, Shanghai Academy of Agricultural Sciences, Shanghai, China; ^2^Joint International Research Laboratory of Metabolic and Developmental Sciences, School of Life Sciences and Biotechnology, Shanghai Jiao Tong University, Shanghai, China

**Keywords:** *Brassica napus* L., rapeseed, transcriptome, genic male sterility, pollen development

## Abstract

Rapeseed is the third leading source of edible oil in the world. Genic male sterility (GMS) lines provide crucial material for harnessing heterosis for rapeseed. GMS lines have been widely used successfully for rapeseed hybrid production. The physiological and molecular mechanism of pollen development in GMS lines of rapeseed (*Brassica napus* L.) need to be determined for the creation of hybrids and cultivation of new varieties. However, limited studies have focused on systematically mining genes that regulate the pollen development of GMS lines in *B. napus*. In the present study, to determine the stage at which pollen development begins to show abnormality in the GMS lines, we performed semi-thin section analysis of the anthers with five pollen development stages. The results indicated that the abnormal pollen development in DGMS lines might start at the meiotic stage, and abnormal pollen development in RGMS lines probably occurred before the tetrad stage. To investigate the critical genes and pathways involved in pollen development in GMS lines, we constructed and sequenced 24 transcriptome libraries for the flower buds from the fertile and sterile lines of two recessive GMS (RGMS) lines (6251AB and 6284AB) and two dominant GMS (DGMS) lines (4001AB and 4006AB). A total of 23,554 redundant DEGs with over two-fold change between sterile and fertile lines were obtained. A total of 346 DEGs were specifically related to DGMS, while 1,553 DEGs were specifically related to RGMS. A total of 1,545 DEGs were shared between DGMS and RGMS. And 253 transcription factors were found to be differentially expressed between the sterile and fertile lines of GMS. In addition, 6,099 DEGs possibly related to anther, pollen, and microspore development processes were identified. Many of these genes have been reported to be involved in anther and microspore developmental processes. Several DEGs were speculated to be key genes involved in the regulation of fertility. Three differentially expressed genes were randomly selected and their expression levels were verified by quantitative PCR (qRT-PCR). The results of qRT-PCR largely agreed with the transcriptome sequencing results. Our findings provide a global view of genes that are potentially involved in GMS occurrence. The expression profiles and function analysis of these DEGs were provided to expand our understanding of the complex molecular mechanism in pollen and sterility development in *B. napus.*

## Introduction

*Brassica napus* L. is an evolutionarily young allopolyploid (AACC) brassicaceous species, which was derived from *Brassica rapa* and *Brassica oleracea* ~7,500 years ago by natural hybridization and chromosome doubling (Wang et al., 2011; Chalhoub et al., 2014). Rapeseed is the third leading source of edible oil in the world. Rapeseed oil has high oleic acid, linoleic acid, and linolenic acid content and with low erucic acid and glucosinolate contents. Through natural and artificial selection, rapeseed has become a multipurpose crop (i.e., vegetable and oil for human; forage crop for livestock) (Allender and King, 2010). China is a supplier of rapeseed and is the oldest country that is involved in the cultivation of rapeseed in the world. The annual rapeseed plantation area and total output of oilseed yield in China have always been at the forefront of the world (Wang et al., 2007). The improvement of yield is mainly attributed to the large-scale application of hybrid rapeseed. More than 60% of rapeseed production involves hybrid rapeseed. The main resource used in heterosis in rapeseed contains genic male sterility (GMS), cytoplasmic male sterility (CMS), self-incompatibility, and chemical male sterility (Kaul, 1988).

GMS contains dominant GMS (DGMS) and recessive GMS (RGMS), which retain complete and stable male sterility. In cabbage (*B. oleracea* var. *capitata*), a male-sterile line (79-399-3) that carries a dominant QTL (*Ms-cd-1*) for male sterility was identified from line 79-399. This QTL causes abnormal callose degeneration and arrest of microspore cell division at the tetrad stage (Lou et al., 2007). Eventually, a novel gene (*Bol357N3*) was identified as a candidate gene for *Ms-cd1* (Liang et al., 2017). In rapeseed, DGMS and RGMS are widely used for hybrid rapeseed production (Fu, 2000). In 2005, Song et al. found multiple alleles in one locus inheritance in the DGMS line 609AB. In this model, *Mf*, *Ms*, and *ms* are three alleles at the same locus, with a relationship of *Mf* > *Ms* > *ms*. The recessive allele is associated with normal fertility. Multiple-allele DGMS is widely used for hybrid rapeseed seed production through the construction of a three-line hybrid system (Song et al., 2006; Liu et al., 2008). In the RGMS system, most inbred lines can restore their fertility and hybrids with strong heterosis are easily bred. In 1998, 9012AB was used in a three-line hybrid production system, and its male sterility was thought to be controlled by three independent genes, namely, *BnMs3*, *BnMs4*, and *BnRf* (Chen et al., 1998). In 2012, Dong et al. demonstrated that the *BnMs4* locus is an actual allele of *BnRf*, which was designated as *BnRf^a^*. The allele from 9012A was designated as *BnRf^b^*, and the allele from temporary maintainer was designated as *BnRf^c^*. *BnRf^a^* was dominant over *BnRf^b^*, and *BnRf^b^* was dominant over *BnRf^c^* (Dong et al., 2012). In total, the DGMS and RGMS lines are valuable resource, and they have been used successfully for rapeseed hybrid production in the world.

As a male gametophyte, pollen participates in sexual reproduction in flowering plants and directly influences seed generation (McCormick, 2004). Pollen development is a complex process that involves the formation of microsporocyte in the anther, male meiosis, and microspores being released from the tetrads, pollen wall development, and pollen maturation. Then, the mature pollen grains are released after anther dehiscence (Gómez et al., 2015). Fertility and pollen development involve many regulatory pathways and related genes. In Arabidopsis, 13,977 male gametophyte-expressed mRNAs were identified using Affymetrix ATH1 genome arrays. Among these mRNAs, 1,355 specific mRNAs were identified as male gametophyte-specific transcripts, including *AtPAB3* (At1g22760), *AtPAB6* (At3g16380), *AtPAB7* (At2g36660), *AteIF2-B3* (At3g07920), *AteIF4G*-like (At4g30680), and *AteIF6-2* (At2g39820) (Honys and Twell, 2004). Two transcriptome factors *AMS* and *AtBZIP34* control pollen wall formation during Arabidopsis anther or pollen development (Sorensen et al., 2003; Gibalova et al., 2009; Xu et al., 2010). Five transcription factors (*MYB80*, *MS1*, *AMS*, *DYT1*, and *TDF1*) regulate tapetum development and further influence the development of microspores by controlling callose dissolution, pollen extine formation, and tapetal programmed cell death (Zhang et al., 2006; Yang et al., 2007; Zhang et al., 2007; Zhu et al., 2008; Gu et al., 2014). In *Brassica campestris*, through comparative transcriptome analysis and ChIP-sequencing, 8,288 genes were differentially expressed in at least one stage of sterile floral buds compared with the fertile buds of *B. campestris*. Among these DEGs, *Bra016531* was associated with tapetum development and function during pollen development (Shen et al., 2019). Moreover, various genes in *B. campestris* are involved in pollen development, such as *BcMF2* (Huang et al., 2009), *BcMF3* (Liu et al., 2006), *BcMF4* (Liu et al., 2006), up to *BcMF23* (Lin et al., 2017). Among these genes, *BcMF6* (Zhang et al., 2008), *BcMF2* (Huang et al., 2009), and *BcMF9* (Huang et al., 2009) are polygalacturonase genes, which participate in pollen extine or intine development. *BcMF21* is involved in pollen extine development and germination (Jiang et al., 2014). In *B. napus*, a global dynamic transcriptome programming of rapeseed anther was performed at different development stages. The results indicated that 35,470 transcripts were expressed in at least one of the anther development stages from the pollen mother cell stage to the mature pollen stage, suggesting that much more genes that are involved in pollen development need to be studied (Li et al., 2016). These findings provide important information for understanding the gene regulatory networks of pollen development. However, the mechanisms of pollen development are still incomplete, and more studies are needed.

In the present study, two RGMS lines (6251AB and 6284AB) and two DGMS lines (4001AB and 4006AB) were used to systematically explore the genes involved in pollen development in GMS lines. First, morphological differences were observed in the flower organs of the fertile and sterile lines. Semi-thin section was conducted to determine the stage at which pollen development begins to show abnormality in the sterile lines. Thereafter, 24 transcriptome libraries were constructed and sequenced for the flower buds from the fertile and sterile lines. DEGs between the fertile and sterile line were screened, and their expression differences were analyzed by transcriptome sequencing. Then, the gene function and related special pathway of the DEGs were analyzed using GO classification and KEGG annotation analysis. Additionally, DEGs that are specifically related to DGMS or RGMS and the shared DEGs involved in both DGMS and RGMS were identified. The differentially expressed TFs were investigated. Screening of DEGs that may be involved in anther and microspore development was conducted and the potential gene functions were analyzed according to the functions of their orthologous Arabidopsis genes. Several potential key DEGs involved in RGMS and DGMS were speculated. These findings would provide a foundation for evaluating the complex molecular mechanisms in pollen development and GMS occurrence in Brassica crops.

## Materials and methods

### Plant materials

The two recessive genic male sterile lines (6251AB and 6284AB) and two dominant genic male sterile lines (4001AB and 4006AB) of *B. napus* were used in this study and planted in the experimental farm of Zhuanghang comprehensive experimental station of Shanghai Academy of Agricultural Sciences. The 6251A and 6284A lines are male sterile lines, and 6251B and 6284B are fertile lines. The 4001A and 4006A lines are male sterile lines, and 4001B and 4006B are fertile lines (Jiang et al., 2021). During flowering stage, floral buds at five developing stages were sampled, which were corresponding five pollen development stages (stage I, pollen mother cell stage; stage II, tetrad stage; stage III, uninucleate microspore stage; stage IV, binucleate microspore stage; and stage V, mature pollen stage) based on the bud size (Huang et al., 2008). The floral buds at five developing stages were harvested from more than 10 plants of the eight lines, including 6251A, 6251B, 6284A, 6284B, 4001A, 4001B, 4006A, and 4006B. Then, the samples were quickly frozen in liquid nitrogen and stored at –80°C. Three independent biological replicates were collected for each kind of sample.

### Flower morphology and cytological observation of GMS lines

At the flowering stage, the four floral organs of a complete flower were observed under a stereomicroscope (Leica, Germany). Twenty flowers from different lines were collected for measurement. The difference was validated by t test (*N*=20, *P*<0.05). The flower buds at five pollen development stages based on the bud size (Huang et al., 2008) were collected and stored in 70% formalin–acetic acid–alcohol fixative solution at 4°C. Semi-thin section observation was performed using the flower buds at five pollen development stages at Wuhan Servicebio Technology Co., Ltd.

### Library construction and illumina sequencing

Total RNA was extracted using TRIzol reagent (Invitrogen, USA). For each kind of sample, three biological replicates were employed. RNA samples were with an OD260/OD280 ratio of 2.0. Mix equal amounts of RNA from flower buds at five developmental stages. A total content of more than 2 µg were qualified for transcription library construction (Lin et al., 2019; Huang et al., 2020). Twenty-four sequencing libraries (three biological replicates each for “6251A”, “6251B”, “6284A”, “6284B”, “4001A”, “4001B”, “4006A”, and “4006B”) were constructed and then sequenced using Illumina Hiseq 2500/Miseq at Beijing Novogene Bioinformatics Technology Co. Ltd.

### Data analysis

The clean reads were obtained by first filtering the reads with adapters, N-containing reads, and low-quality reads. Then, the sequencing error rate was checked. Lastly, the GC content distribution was checked. Then, the clean reads were mapped to the *B. napus* genome (http://brassicadb.agridata.cn/brad/) using HISAT2 software (Kim et al., 2015). The distribution of all the reads in genome was analyzed. The novel genes were predicted, and all the gene expression values (FPKM) were analyzed based on the read counts on genome. Pearson correlation analysis between samples was adopted to examine the reliability of experiment and rationality of sample selection. After the gene expression was quantified, the raw readcount was normalized. The *p* value and false discovery rate (FDR) were calculated using the DESeq2 software (Love et al., 2014). The readcount after normalization (FPKM) was used to screen the DEGs between different samples. *P* value < 0.05, FDR < 0.01, and |log_2_(FoldChange)| > 1 based on three biological replicates were considered as DEGs. The online software of Venny 2.1.0 was used to generate the Venn diagrams. To explore the related biological function or pathway of DEGs, gene ontology (GO) enrichment analysis of DEGs was implemented using the clusterProfiler R package, in which gene length bias was corrected. The FDR < 0.05 was considered as significantly enriched. KEGG is a database resource for understanding high-level functions and utilities of the biological system (http://www.genome.jp/kegg/). ClusterProfiler R package was used to test the statistical enrichment of DEGs in KEGG pathways. FDR < 0.05 was defined as significant differential expression.

### QRT-PCR

Total RNA (2µg) was reverse-transcribed into cDNA by using reverse transcriptase (TaKaRa) with oligo (dT). The specific primers used in qPCR are listed in **Table S8**. The qRT-PCR reactions were conducted in the CFX96 real-time system (Bio-Rad, Hercules, CA, USA) by using ChamQ Universal SYBR qPCR Master Mix (Vazyme, China). The 20 µL reaction mixture contained 0.5 µL of cDNA, 7.9 µL of ddH_2_O, 10 µL of 2X SYBR mixture, 0.8 µL of forward primer, and 0.8 µL of reverse primer. Three biological replicates were employed with three technical replicates for each sample, and the relative expression levels were quantified using the 2^−ΔΔCt^ method (Livak and Schmittgen, 2001).

## Results

### Morphological and cytological characterization of the fertile and sterile lines

**Figure 1:**
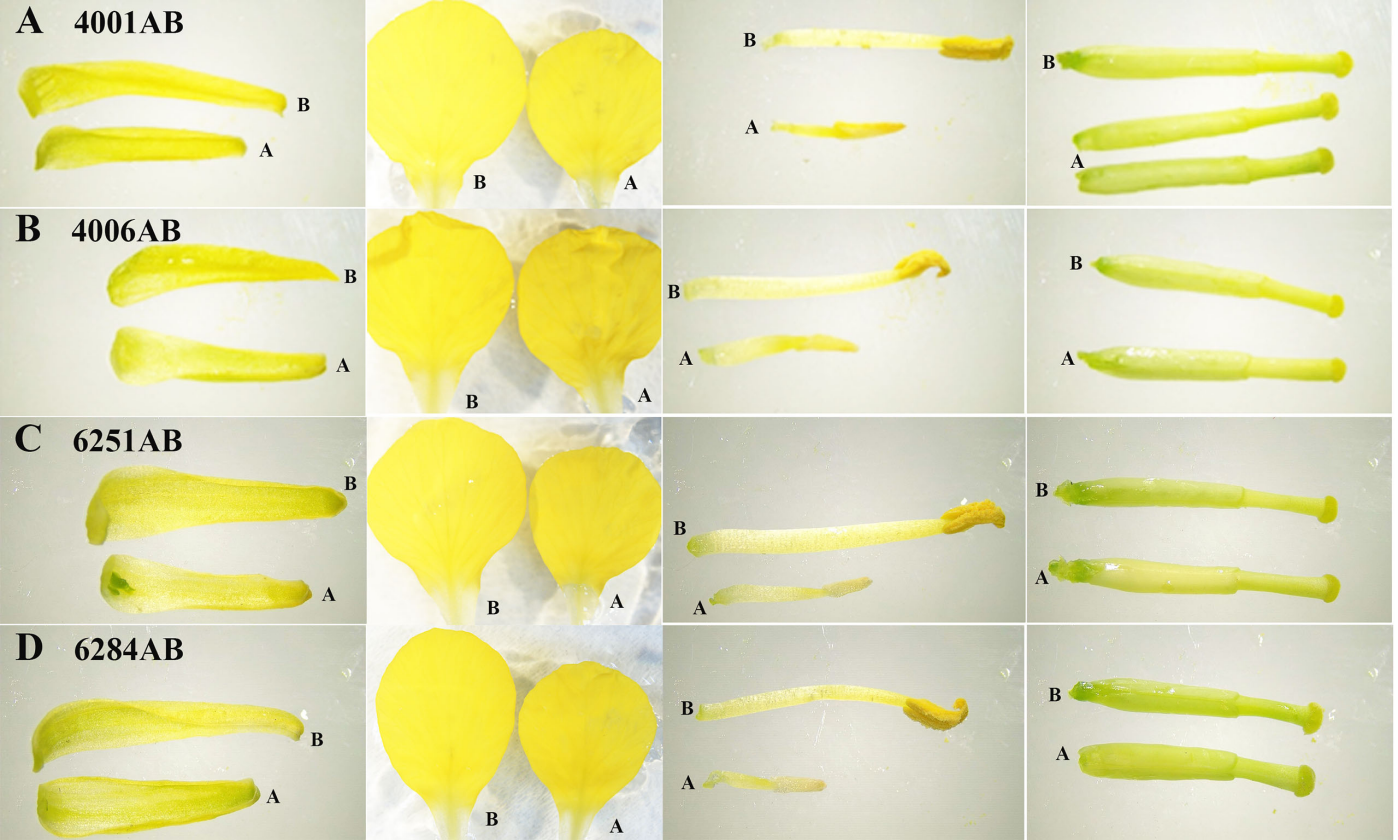
Phenotype observation of flower organs from 4001AB, 4006AB, 6251AB, and 6284AB. **(A)**, flower organs of 4001AB; **(B)**, flower organs of 4006AB; **(C)**, flower organs of 6251AB; and **(D)**, flower organs of 6284AB. The four types of flower organs were sepals, petals, stamens, and pistils. The “A” and “B” letters marked next to the flower organs indicate the organs from A or B line.

**Figure 2:**
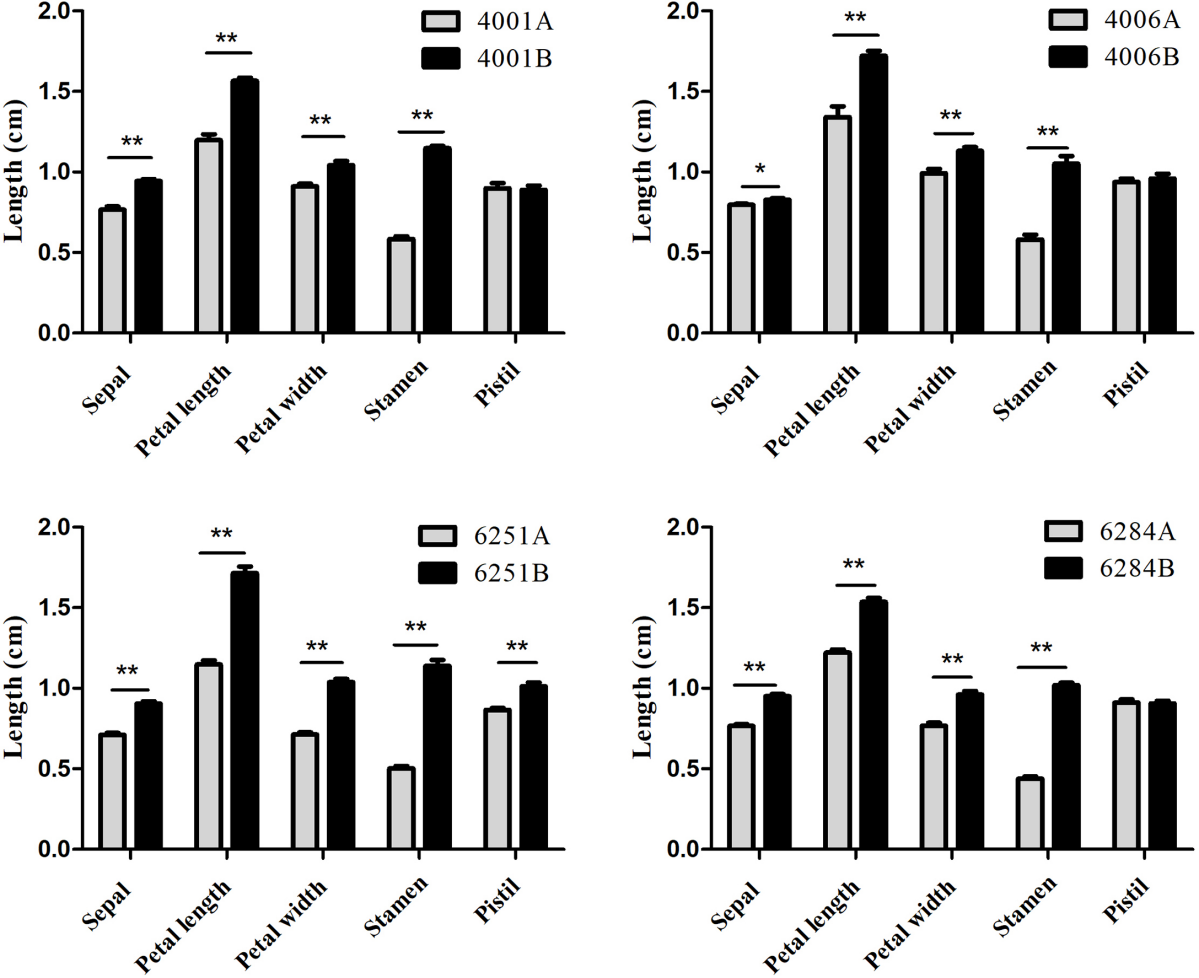
Average lengths of floral organs from 4001AB, 4006AB, 6251AB, and 6284AB. Error bars represent SD. The values in the figure are expressed as the means ± SD from 20 repeats. Differences between A and B line were considered significant (*) if p < 0.05 (Student’s t-test) and highly significant (**) if p < 0.01 (Student’s t-test).

The DGMS (4001AB, 4006AB) and RGMS (6251AB, 6284AB) lines were planted in the field under the same environment. The plant phenotypes of the A and B lines were observed along with their development processes. No significant difference was observed in the growth status during the vegetative growth among different GMS lines. For each GMS line, the flower development in A line was abnormal compared with that in B line (**Figure 1**). Five main traits of flower organs were examined at representative stages, including sepal length, petal length, petal width, stamen length, and pistil length. For all the four GMS materials (4001AB, 4006AB, 6251AB, and 6284AB), the A lines had shorter sepal, petal, stamen, and narrower petal than the B line. In 6251AB, the A line pistils were shorter, while in other GMS materials, the pistil lengths were not significantly different between the A and B lines (**Figure 2**).

**Figure 3:**
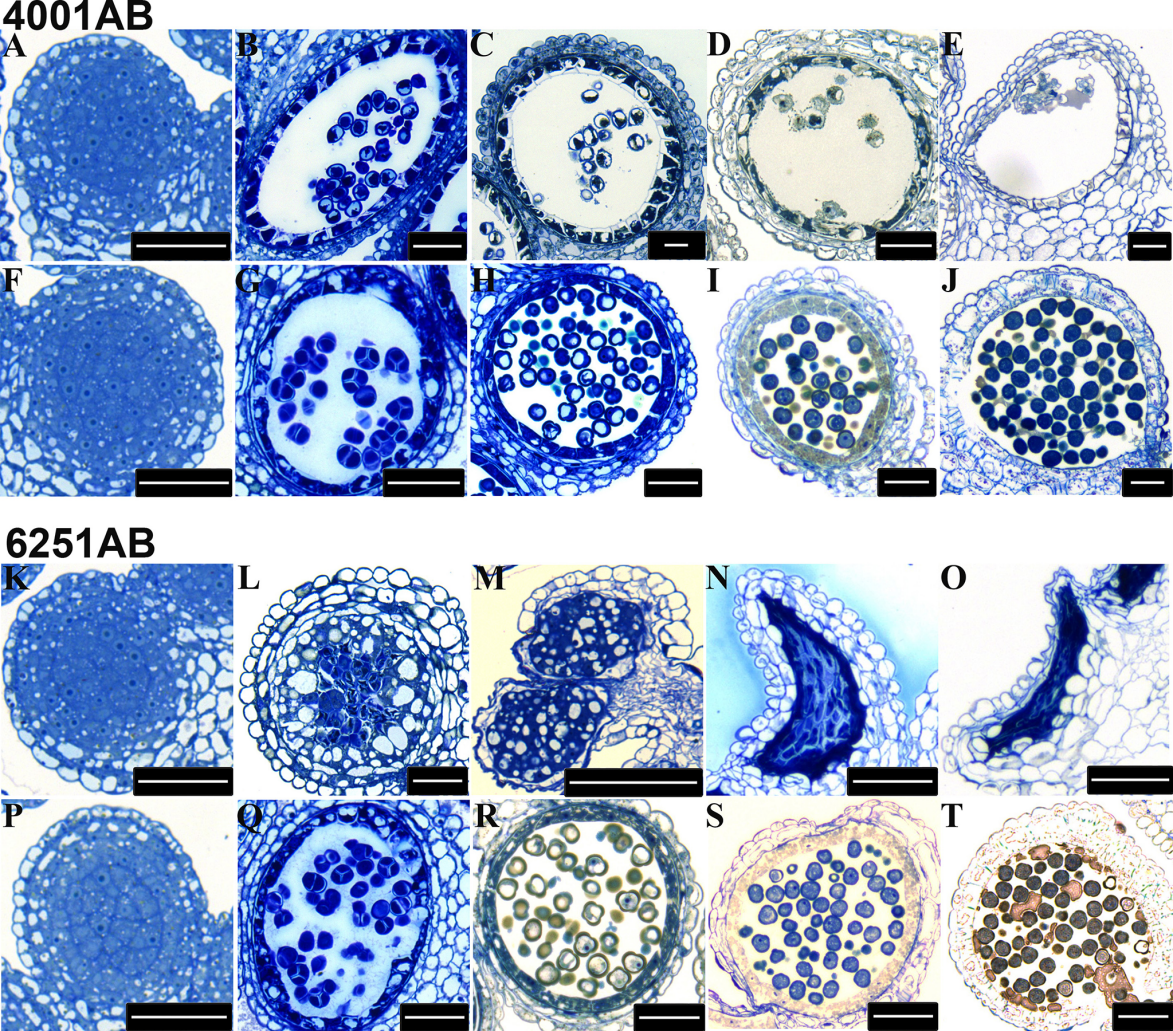
Transverse sections of anthers from the 4001AB and 6251AB lines. The semi-thin sections of anthers from 4001A **(A–E)** and 4001B **(F–J)** are shown above. The semi-thin sections of anthers from 6251A **(K–O)** and 6251B **(P–T)** are shown below. The microspores at the pollen mother cell stage **(A, F, K,** and **P)**, tetrad stage **(B, G, L,** and **Q)**, uninucleate microspore stage **(C, H, M,** and **R)**, binucleate microspore stage **(D, I, N,** and **S)**, and trinucleate microspore stage **(E, J, O,** and **T)** were observed. Scale bars were 50 µm.

To determine the stage at which the abnormality of pollen development began in the GMS lines, we chose 4001AB and 6251AB, which represent the DGMS and RGMS lines, respectively, and conducted semi-thin section analysis of anthers at five pollen development stages. For 4001AB, no difference and abnormality were detected between 4001A and 4001B at the pollen mother cell stage (**Figures 3A, F**). At the tetrad stage, 4001B could form normal tetrads, but 4001A did not (**Figures 3B, G**). At the uninucleate and binucleate microspore stages, the microspores in 4001B were round and full, showing one and two obvious nucleates, respectively (**Figures 3H, I**). At the trinucleate microspore stage, all the microspores in 4001B could form normal mature pollen grains (**Figure 3J**). However, in 4001A, the tetrad stage and later stages were not observed. The abnormal pollen mother cells were gradually degraded, and eventually only a little inclusion remained in the pollen sac of 4001A (**Figures 3B, C, D, E**). Therefore, abnormal pollen development in 4001A might start at the meiotic stage.

For 6251AB, no abnormality was detected in the A and B line at the pollen mother cell stage (**Figures 3K, P**). At the tetrad stage, 6251B could form normal tetrads (**Figure 3Q**). While no tetrads were observed in 6251A, and the tapetal cells were aberrant, swollen and had excess vacuolization (**Figure 3L**). And the cell contents were messed up in pollen sac (**Figure 3L**). At the uninucleate microspore stage, the microspores in 6251B developed normally (**Figure 3R**), while the anther cells showed structural defects and the tapetal cells were degraded and messed up with other degradants in 6251A (**Figure 3M**). At the binucleate and trinucleate microspore stage, the anthers shrunk seriously and stained into dark blue with degradants (**Figures 3N, O**). In 6251B, the anthers and microspores were round and full. The microspores developed well and eventually formed normal mature pollen grains (**Figures 3S, T**).Therefore, abnormal pollen development in 6251A might occur before the tetrad stage.

### Transcriptome sequencing and correlation analysis

To identify mRNAs related to pollen and fertile development in DGMS and RGMS lines, transcriptome sequencing was conducted. The flower buds from the sterile (4001A, 4006A) and fertile (4001B, 4006B) lines were collected. The flower buds of the sterile (6251A, 6284A) and fertile (6251B and 6284B) lines were also collected. Three biological replicates were employed for each sample. In total, 24 cDNA libraries were prepared for transcriptome sequencing. As shown in **Table 1**, 186 G clean bases were obtained, and the clean bases of each library ranged from 5.88 G to 9.46 G with Q30 > 92.86% and GC > 44.41%. The clean reads were then mapped to the *B. napus* reference genome (http://brassicadb.cn/) with a mapping rate of 89.73%–91.07%. The mapping rate of the unique reads ranged from 86.23% to 87.32%, while the mapping rate of the multi-mapped reads was less than 5% (**Table 1**). To visualize the underlying structure of the RNA-seq data and emphasize the high degree of correlation between biological replicates, principal component analysis (PCA) and Pearson correlation were performed using the fragments per kilobase of transcript per million mapped reads (FPKM) value. Excellent correlations were observed between A and B lines for each GMS material (**Figure S1A**). Moreover, PCA analysis show that the data collected from the fertile and sterile lines of DGMS and RGMS lines could be clearly separated. The three biological replications were concentrated well (**Figure S1B**). Therefore, the RNA-seq data were considered reliable for subsequent analysis.

**Table 1:** The alignment statistics result with the reference genome for all samples.

sample	clean_reads	clean_bases	Q30	GC_pct	total_map	unique_map
6284A1	45948332	6.89G	92.86	45.3	41362911 (90.02%)	39667573 (86.33%)
6284A2	62088566	9.31G	93.88	45.24	56097765 (90.35%)	53793729 (86.64%)
6284A3	57424402	8.61G	93.58	45.33	52102792 (90.73%)	49971574 (87.02%)
6284B1	39172350	5.88G	93.51	45.31	35631954 (90.96%)	34073401 (86.98%)
6284B2	51054958	7.66G	93.91	45.06	46436011 (90.95%)	44363635 (86.89%)
6284B3	55454398	8.32G	93.31	45.13	50277612 (90.66%)	48127070 (86.79%)
6251A1	56355344	8.45G	93.76	45.82	51289259 (91.01%)	49211344 (87.32%)
6251A2	48132688	7.22G	93.47	45	43331171 (90.02%)	41606066 (86.44%)
6251A3	59591480	8.94G	94.21	45.39	54021703(90.65%)	51904671(87.1%)
6251B1	41147282	6.17G	94.09	45.52	37455212 (91.03%)	35888303 (87.22%)
6251B2	48823854	7.32G	94.19	45.12	44199783 (90.53%)	42403466 (86.85%)
6251B3	60574856	9.09G	93.39	45.46	55133378 (91.02%)	52723959 (87.04%)
4001A1	47587290	7.14G	93.73	45.38	43222164 (90.83%)	41487710 (87.18%)
4001A2	44763242	6.71G	94.18	45.1	40600371 (90.7%)	38857683 (86.81%)
4001A3	56108092	8.42G	94.09	45.3	50858746 (90.64%)	48822643 (87.02%)
4001B1	54646558	8.2G	94.07	45.18	49625443 (90.81%)	47607811 (87.12%)
4001B2	55660646	8.35G	93.88	45.09	50446058 (90.63%)	48181473 (86.56%)
4001B3	47664592	7.15G	93.78	45.18	43406941 (91.07%)	41609392 (87.3%)
4006A1	50508724	7.58G	94.02	44.6	45406051 (89.9%)	43596855 (86.32%)
4006A2	46000888	6.9G	93.89	44.41	41278231 (89.73%)	39667726 (86.23%)
4006A3	51603244	7.74G	94.1	44.67	46353841 (89.83%)	44509998 (86.25%)
4006B1	55926718	8.39G	93.99	44.46	50375527 (90.07%)	48348252 (86.45%)
4006B2	40630008	6.09G	94.32	44.73	36936999 (90.91%)	35457501 (87.27%)
4006B3	63088340	9.46G	94.01	44.66	57140191 (90.57%)	54743421 (86.77%)

Sample: sample name; clean_reads: the number of clean reads, the single-ended meter; clean_bases: the number of clean data; Q30: Q-score of clean data ≥30; GC_pct: the percentage of GC-content in clean data; total_map: the number of reads mapped to the reference genome and its percentage in celan reads; unique_mapped reads: the number of reads mapped to the only location of the reference genome and its percentage in clean reads.

### Differentially expressed genes analysis

**Figure 4:**
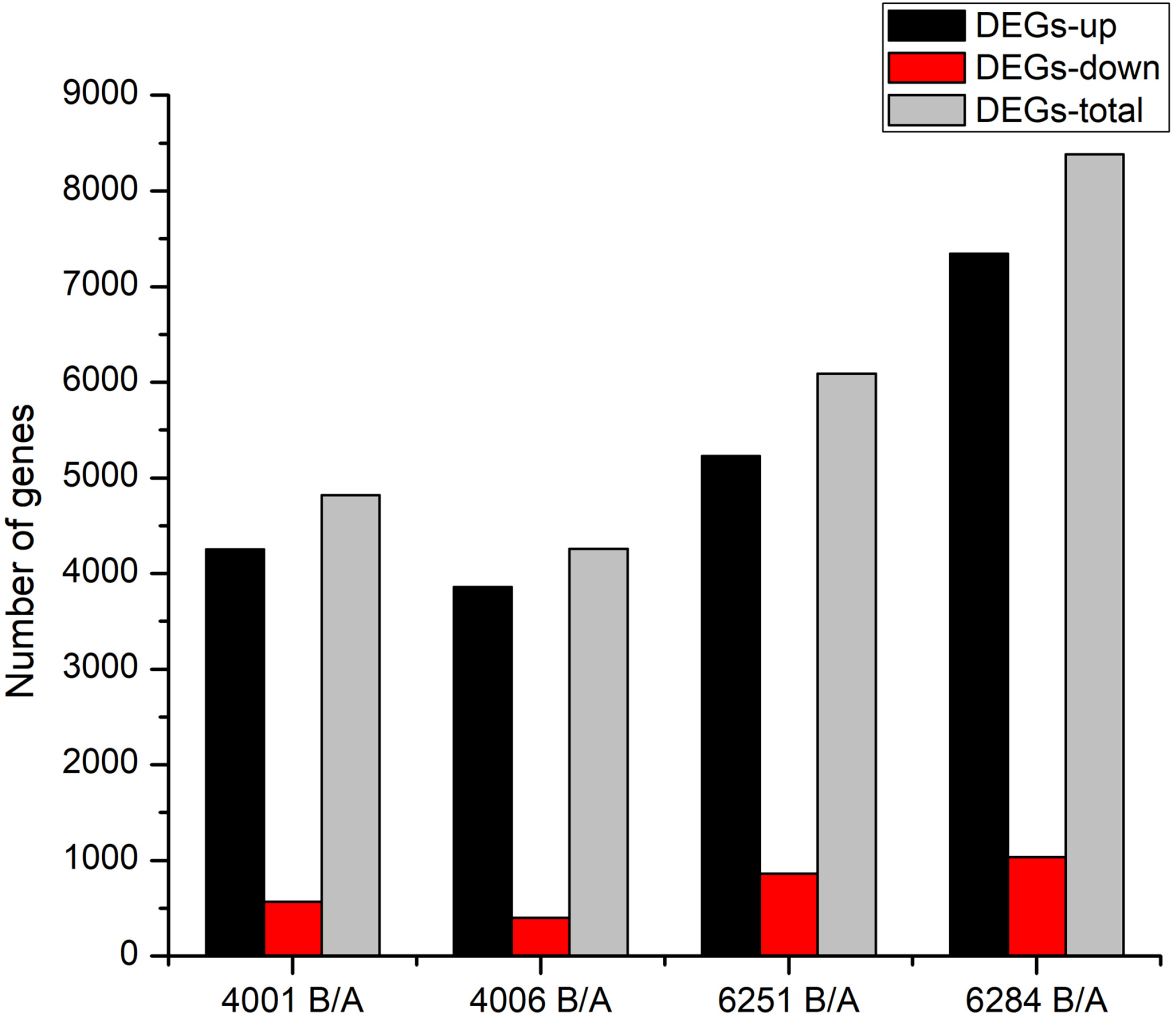
Column diagram shows the numbers of DEGs in four GMS materials. The dark and red columns represent the up- and downregulated DEGs in B lines. The gray columns represent the total DEGs in the corresponding GMS material.

**Figure 5:**
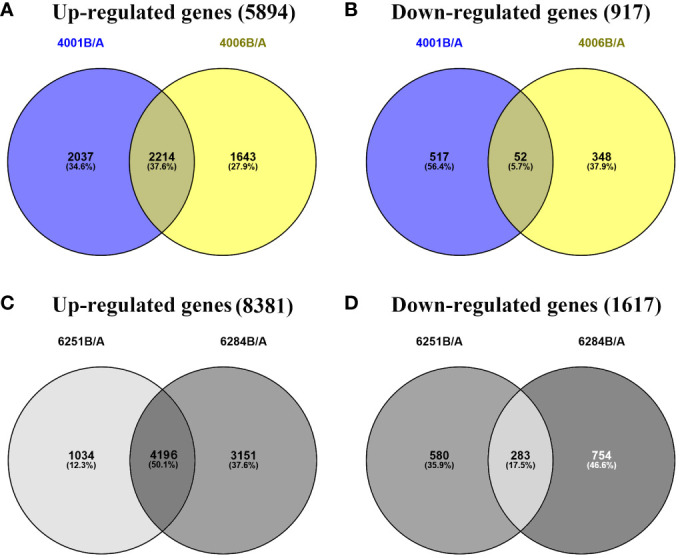
Venn diagrams representing the numbers of DEGs and the overlaps of sets obtained across four comparisons. The up- **(A)** and downregulated genes **(B)** in DGMS. The up- **(C)** and downregulated genes **(D)** in RGMS.

**Figure 6:**
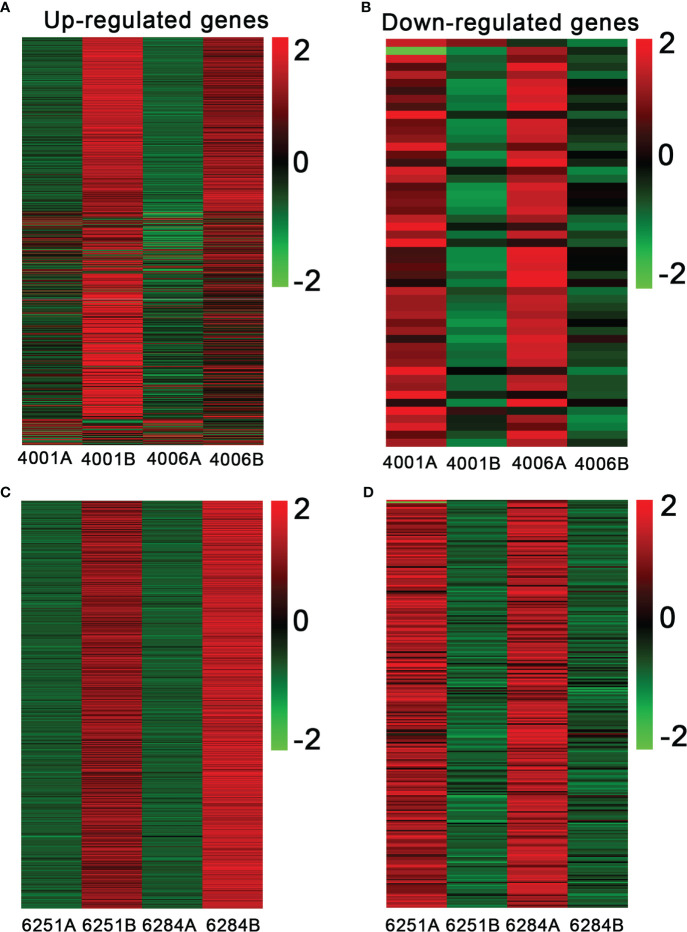
Heatmap showing the expression patterns of differentially expressed genes in the fertile and sterile lines. The up- **(A)** and downregulated genes **(B)** in DGMS. The up- **(C)** and downregulated genes **(D)** in RGMS. The legend represents the fold change level.

The FPKM value of transcripts was used for identifying DEGs (|log_2_ (fold change)| ≥1, *q* value < 0.05 and FDR< 0.01) between the fertile and sterile lines (4001B vs. 4001A, 4006B vs. 4006A, 6251B vs. 6251A, and 6284B vs. 6284A). Finally, 23,554 redundant DEGs were obtained. Among these DEGs, 4,820 DEGs were obtained in 4001AB, including 4,251 upregulated genes and 569 downregulated genes in 4001B, compared with 4001A. In total, 4,257 DEGs were obtained in 4006AB, including 3,857 upregulated genes and 400 downregulated genes in 4006B, compared with 4006A. In addition, 6,093 DEGs (5,230 upregulated and 863 downregulated) and 8,384 DEGs (7,347 upregulated and 1,037 downregulated) were obtained in 6251AB and 6284AB, respectively (**Figure 4**; **Table S1**). The number of upregulated genes was higher than that of downregulated genes. To explore the DEGs involved in the DGMS and RGMS lines, we conducted a Venn diagram analysis. Based on the comparison between 4001AB and 4006AB, 2,214 (37.6%) genes were commonly upregulated, and 52 (5.7%) genes were commonly downregulated (**Figures 5A, B**). In addition, 4,196 (50.1%) genes were commonly upregulated, and 283 (17.5%) genes were commonly downregulated in the two RGMS lines (6251AB and 6284AB) (**Figures 5C, D**). To intuitively show the differences and similarities of the DEGs in flower buds from different GMS lines, we performed hierarchical clustering to represent the expression of DEGs (**Figure 6**; **Table S2**). The results showed a significant difference in gene expression profile among the four GMS groups. Most of the DEGs were obviously upregulated in B lines. Some genes were barely expressed in A lines, but they were highly expressed in B lines (**Figure S2A**). Therefore, a large number of DEGs may play a positive regulatory role in pollen development.

### GO function and KEGG pathway analysis of differentially expressed gene

**Figure 7:**
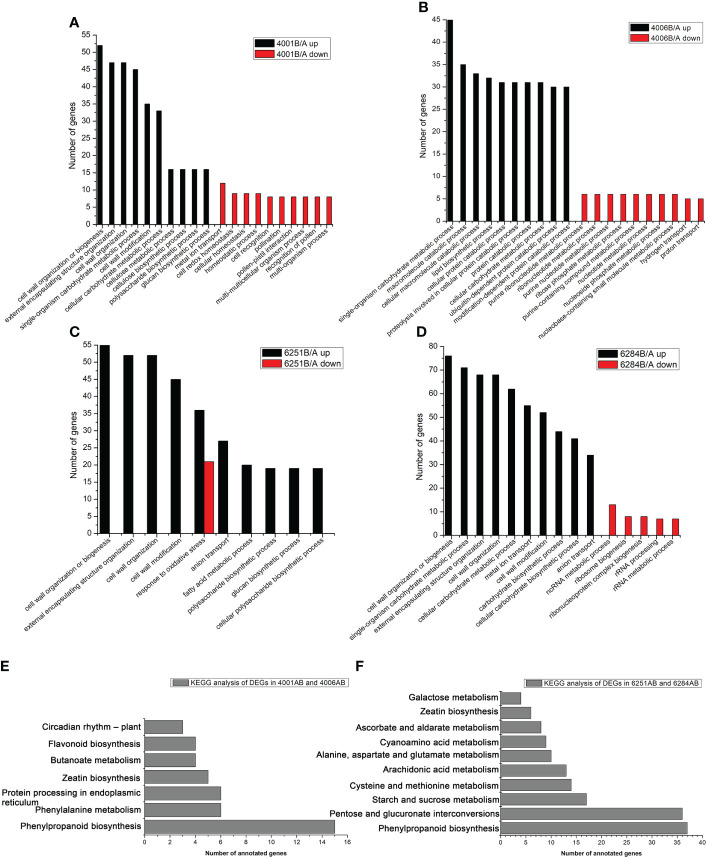
GO and KEGG classification of differentially expressed genes. The enriched GO categories of DEGs in 4001AB **(A)**, 4006AB **(B)**, 6251AB **(C)**, and 6284AB **(D)**. KEGG pathway analysis of DEGs in DGMS **(E)** and RGMS **(F)** based on the KEGG data.

In the present study, an FDR of < 0.05 was used to pick significantly enriched GO terms. The annotated genes were divided into three major functional categories, namely, biological processes (BP), cellular components (CC), and molecular functions (MF, **Table S3**). The main GO terms were significantly enriched in biological processes. For the DEGs in DGMS (4001AB, 4006AB), the GO terms were significantly enriched in cell wall organization or biogenesis (GO: GO:0071554), external encapsulating structure organization (GO: GO:0045229), cell wall organization (GO: GO:0071555), single-organism carbohydrate metabolic process (GO:0044723), macromolecule catabolic process (GO:0009057), cellular macromolecule catabolic process (GO:0044265), and lipid biosynthetic process (GO:0008610, **Figures 7A, B**). Interestingly, for the down-regulated DEGs in 4001B, some GO terms related to pollen development, pollination, and fertilization were significantly enriched, including pollination (GO:0009856), pollen-pistil interaction (GO:0009875), and recognition of pollen (GO:0048544) (**Figure 7A**). The relevant genes included *SDR1* (BnaA07g25970D), *ARK2* (BnaA07g25960D), *RKS2-1* (BnaC08g42100D), *RKS2-2* (BnaC08g42090D), and *SD1-29* (BnaC01g29480D, **Table S3**). For the DEGs in RGMS (6251AB, 6284AB), the GO terms were mainly enriched in cell wall organization or biogenesis (GO:0071554), external encapsulating structure organization (GO:0045229), cell wall organization (GO:0071555), cell wall modification (GO:0042545), and single-organism carbohydrate metabolic process (GO:0044723, **Figures 7C, D**).

KEGG pathway analysis was conducted to investigate whether the DEGs were involved in special pathways. For all the four GMS lines, the most significantly enriched pathway was phenylpropanoid biosynthesis (ath00940). For DGMS lines (4001AB and 4006AB), the most significantly enriched pathway was phenylpropanoid biosynthesis (ath00940), followed by phenylalanine metabolism (ath00360), protein processing in endoplasmic reticulum (ath04141), zeatin biosynthesis (ath00908), and butanoate metabolism (ath00650, **Figure 7E**). For RGMS lines (6251AB and 6284AB), the most significantly enriched pathways were highlighted in phenylpropanoid biosynthesis (ath00940), pentose and glucuronate interconversions (ath00040), starch and sucrose metabolism (ath00500), cysteine and methionine metabolism (ath00270), and arachidonic acid metabolism (ath00590) (**Figure 7F**). Based on the comparison between DGMS and RGMS, two KEGG terms were shared, namely, phenylpropanoid biosynthesis (ath00940) and zeatin biosynthesis (ath00908, **Table S4**). Therefore, a subset of genes potentially played roles in both DGMS and RGMS lines.

### Identification of DEGS specifically related to DGMS or RGMS

In the present study, a subset of DEGs was obtained between the A and B line of DGMS lines. However, these genes did not show differential expression between the A and B line in RGMS. These genes were identified as DGMS-related genes, which might specifically function in DGMS. A total of 346 genes were DGMS-related genes, such as BnaC07g11890D (*HRS1 HOMOLOG3*, *HHO3*) and BnaA02g29090D (**Figures S2B, C**). According to the similar screening method as above, 1,553 genes were identified as RGMS-related genes, such as BnaA03g22110D and BnaA07g32020D. Both of the two genes were differentially expressed between the A and B line in RGMS, but they showed similar expression levels between the A and B line in DGMS (**Figures S2D, E**).

### Identification of shared DEGs related to both DGMS and RGMS

**Figure 8:**
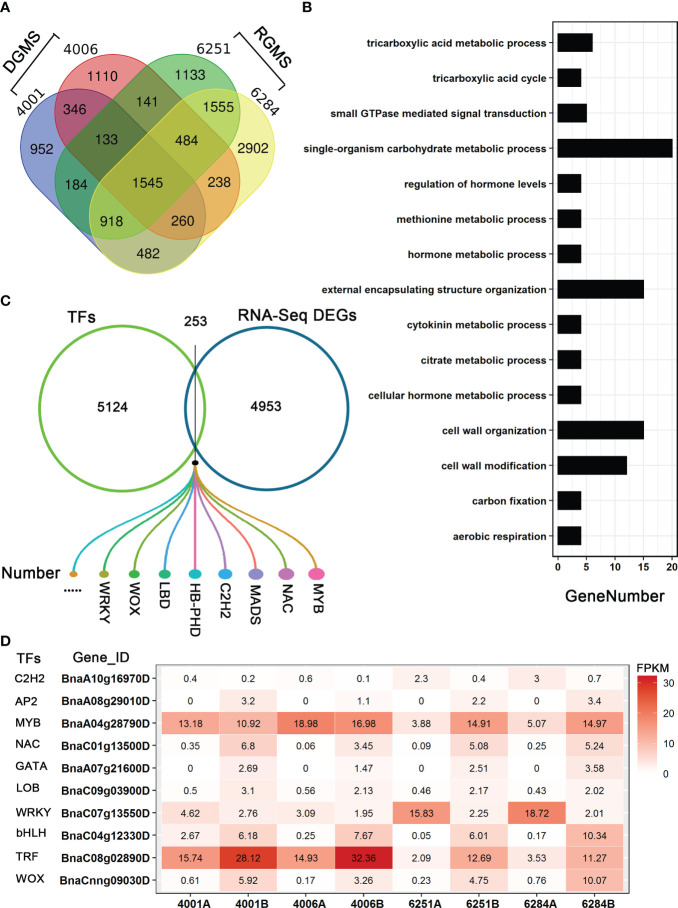
Analysis of DEGs in GMS. **(A)** The Venn diagram shows the overlap of DEGs in RGMS and DGMS. **(B)** GO classification of shared DEGs between RGMS and DGMS. **(C)** Differentially expressed TFs. **(D)** Ten differentially expressed TFs, which were identified from RNA-seq data. Numbers in each box indicate the FPKM values.

**Figure 9:**
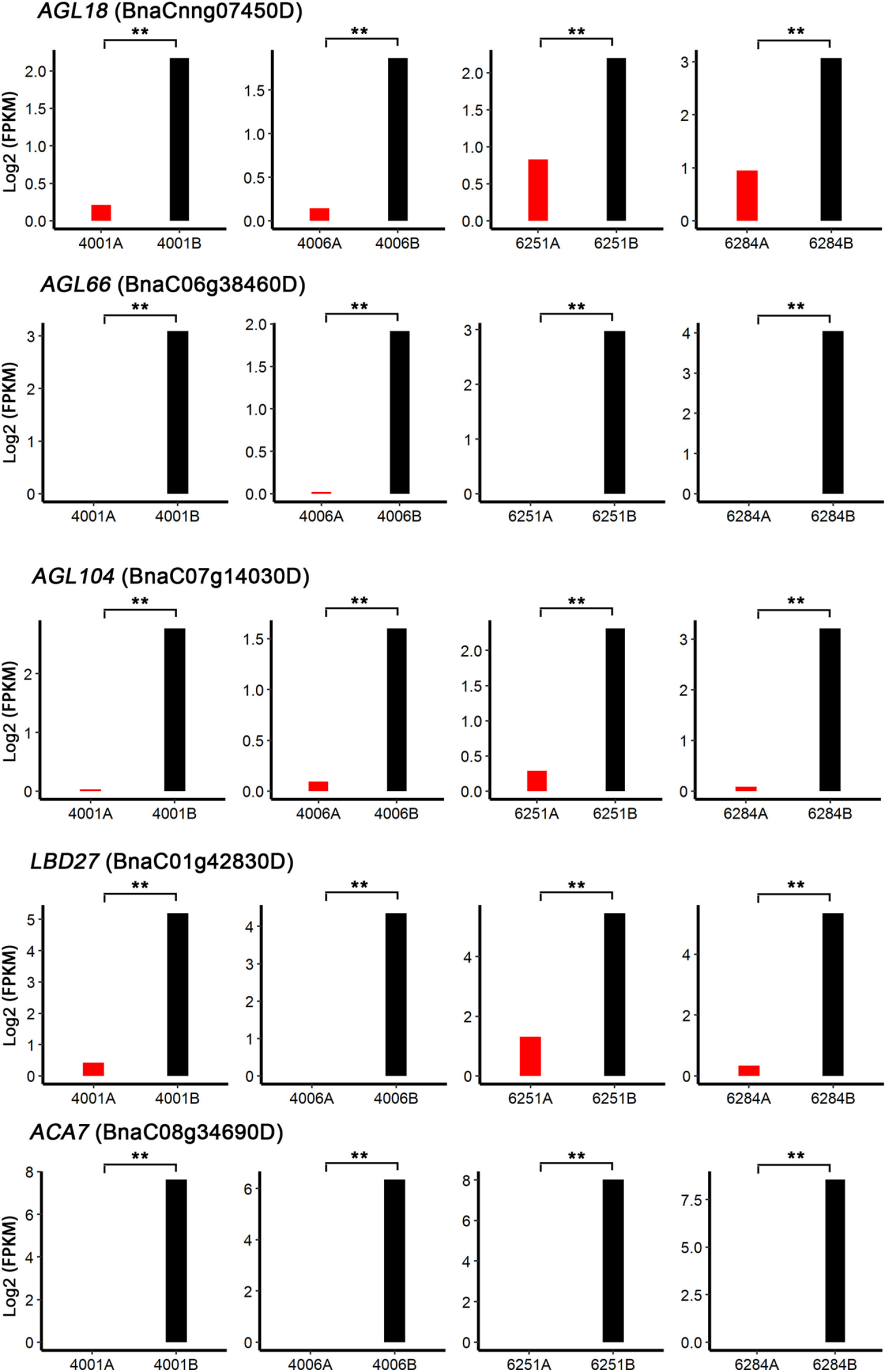
Shared DEGs possibly involved in anther and microspore development. *AGL18*, *AGL66*, *AGL104*, *LBD27*, and *ACA7* were selected to calculate their expression levels by using FPKM values. Significant differences were calculated by performing paired t-test between FPKM values of A and B lines. **P < 0.01.

To identify the candidate genes involved in both DGMS and RGMS, we compared the DEGs identified in DGMS (4001AB and 4006AB) and RGMS (6251AB and 6284AB). In total, 1,545 DEGs were shared between DGMS and RGMS (**Figure 8A**). GO enrichment analysis of the 1,545 DEGs showed that they were mainly enriched in single-organism carbohydrate metabolic process (GO:0044723), external encapsulating structure organization (GO:0045229), and cell wall organization (GO:0071555, **Figure 8B**). To display the expression differences of the shared DEGs in RGMS and DGMS, we selected eight genes and calculated their expression levels by using FPKM values, which were *AGL18*, *AGL66*, *AGL104*, *LBD27*, *ACA7*, *BnaAnng17790D*, *BnaC08g05830D*, and *BnaA07g33840D* (**Figures 9** and **S3**).

### Identification of differentially expressed TFs

To explore the differentially expressed TFs in GMS lines, we downloaded all the *B. napus* transcription factors from BnTIR (http://yanglab.hzau.edu.cn/bntir), and they were compared to all the DEGs. As a result, 253 differentially expressed TFs were found, and they belong to several gene families, such as C2H2, WOX, LBD, WRKY, MYB, and NAC (**Figures 8C, D**; **Table S5**). As DEGs, some had higher expression levels in A lines than in B lines. For example, BnaC07g13550D (a WRKY family TF) was significantly differentially expressed between the A and B lines in the four GMS lines, and much more transcripts accumulated in the A lines (**Figure 8D**). In addition, 48 MYB TFs were differentially expressed between A and B lines, and the proportion (48/253) was larger than the other TFs. BnaA04g28790D was an MYB TF with significantly different expression level in 6251AB and 6284AB lines (**Figure 8D**). Some transcription factors may be involved in the regulation of anther and microspore development.

### Analysis of potential key DEGs involved in GMS

**Figure 10:**
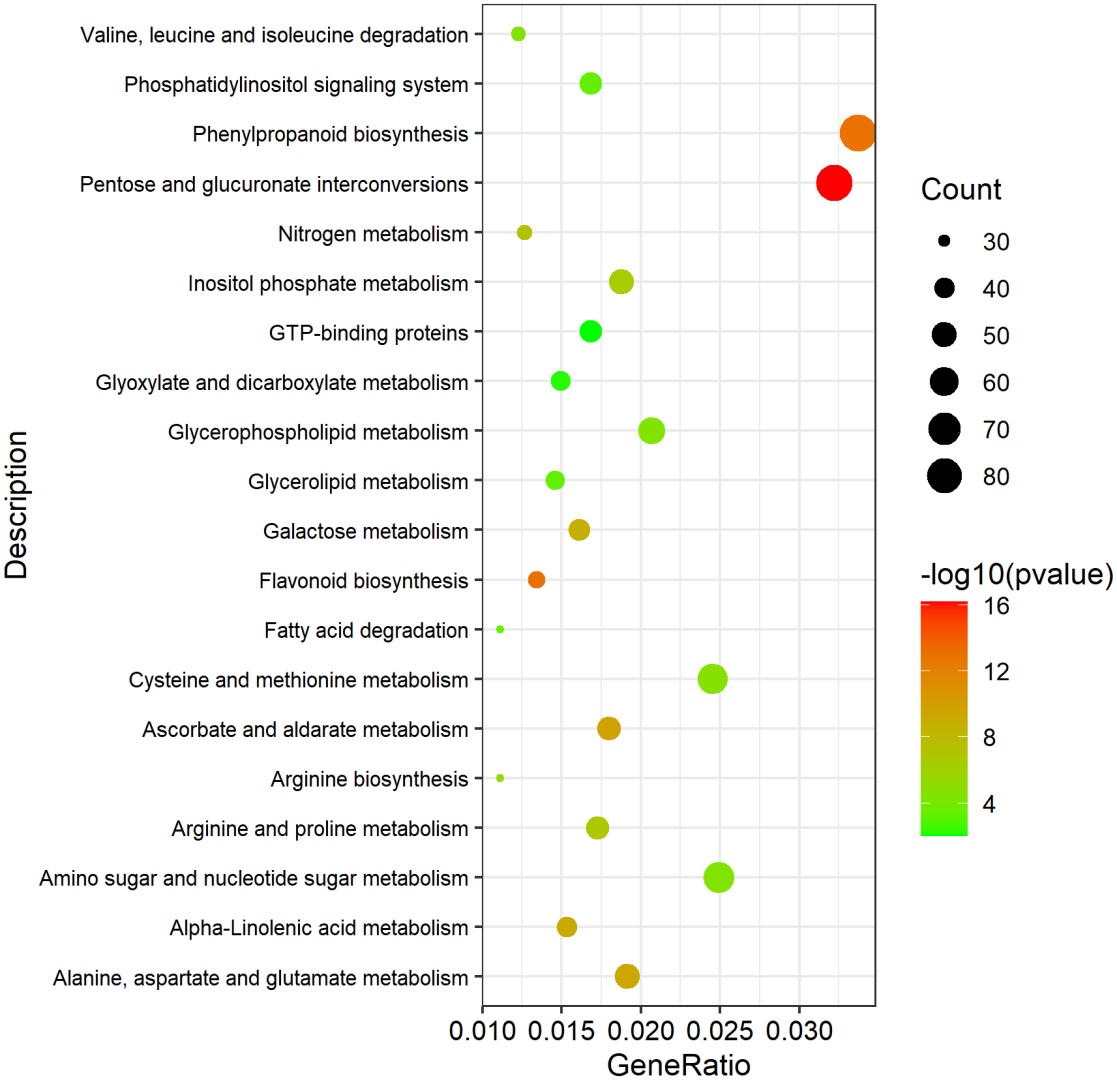
The Top 20 significantly enriched KEGG pathways of the 1073 DEGs possibly involved in pollen development. A total of 1073 DEGs were enriched in 27 significantly pathways (p-value <0.05, p-adjusted <0.05, q-value <0.05). The Top 20 pathways were used to draw the KEGG map.

To investigate the potential DEGs related to pollen development processes, 13,031 Arabidopsis genes related to anther, pollen, and microspore development were downloaded from TAIR website (https://www.arabidopsis.org/index.jsp). And 6,099 homologous DEGs in *B. napus* were identified, which might be involved in anther, pollen and microspore development (**Table S6**). KEGG enrichment analysis of these 6,099 DEGs was performed. A total of 1,073 DEGs were enriched in 27 significantly pathways (p-value <0.05, p-adjusted <0.05, q-value <0.05) (**Table S7**). The Top 20 pathways were used to draw the KEGG map (**Figure 10**). In **Table 2**, we excluded genes with lower expression in advance, and listed 74 highly credible candidate genes related to GMS. Their homologous genes in Arabidopsis have been reported in previous studies to be involved in anther and microspore developmental processes, such as anther cell differentiation, tapetum development, pollen mother cell and microspore division, meiosis process, sporopollenin biosynthesis, pollen exine formation, and pollen maturation process. Based on the functions of these Arabidopsis genes, we speculated the 74 DEGs might be also involved in the anther and microspore developmental processes.

**Table 2:** Potential functional analysis of DEGs possibly involved in anther and microspore development.

Classification	DEGs (*Brassica napus*)					*Arabidopsis thaliana*			References
	Locus	Log_2_FoldChange				Locus	Gene name	Functional Description	
		4001B/A	4006B/A	6251B/A	6284B/A				
Anther cell differentiation	BnaA10g23720D	/	/	/	-1.60	AT5G07280	*EMS1, EXS*	A putative leucine-rich repeat receptor protein kinase that controls somatic and reproductive cell fates in Arabidopsis anther. EMS1 controls anther cell differentiation.	Zhao et al., 2002; Huang et al., 2016
	BnaA03g50230D	/	/	1.38	/	AT4G30520	*CIK3*	Encodes SARK (senescence-associated receptor-like kinase). It is one of a group of LRR-RLKs, designated as CIKs.	Cui et al., 2018
	BnaA01g06550D	/	/	/	1.35	
	BnaA10g04110D	/	1.61	1.74	/	AT1G06170	*BHLH089*	Basic_helix-loop-helix_(bHLH)_DNA-binding_superfamily_protein	Cui et al., 2016
	BnaA05g11510D	/	/	2.12	2.46	AT2G31210	*BHLH091*	Basic_helix-loop-helix_(bHLH)_DNA-binding_superfamily_protein	Cui et al., 2016
Tapetum development	BnaA03g23380D	/	-4.22	/	/	AT5G50260	*CEP1*	Encodes a papain-like cysteine protease involved in tapetal programmed cell death and pollen development.	Zhang et al., 2014
	BnaC07g05950D	/	/	1.86	1.12	AT2G16910	*AMS*	Encodes a basic helix-loop helix transcription factor involved in tapetal cell development. Loss of function mutations are male sterile.	Xu et al., 2010
	BnaC03g46740D	/	-1.31	/	/
	BnaA07g03340D	/	/	2.22	1.50
	BnaC02g12480D	/	/	/	-1.38	AT5G56110	*MS188, MYB103, MYB80*	Encodes a member of the R2R3 MYB transcription factor gene family that is required for anther development by regulation tapetum development, callose dissolution and exine formation.	Zhang et al., 2007; Lu et al., 2020
	BnaAnng24260D	/	/	/	-2.43	AT4G21330	*DYT1*	Encodes a bHLH transcription factor strongly expressed in the tapetum. dyt1 mutant exhibits abnormal anther morphology beginning at anther stage 4.	Zhang et al., 2006
	BnaC01g12730D	-2.95	/	/	-1.90
	BnaC06g01060D	-1.44	/	1.31	/	AT1G44970	*PRX9*	Encodes a class III peroxidase that is genetically redundant with PRX40. PRX9 and PRX40 are extensin peroxidases essential for maintaining tapetum and microspore cell wall integrity during Arabidopsis anther development.	Jacobowitz et al., 2019
	BnaA10g28250D	-1.78	/	/	/
	BnaA01g17520D	-1.30	/	/	/	AT4G16270	*PRX40*	Encodes a class III peroxidase that is genetically redundant with PRX9.	Jacobowitz et al., 2019
	BnaC01g21870D	-1.44	/	/	/
	BnaC09g41260D (MS3)	/	/	9.99	8.86	AT5G16620	*ATTIC40*	chloroplast protein import (Tic40)	Li et al., 2012
	BnaA01g00190D (Rf)	/	/	-1.09	-1.86	AT4G37910	*MTHSC70-1*	mitochondrial heat shock protein 70-1	Dong et al., 2012; Deng et al., 2016
	BnaC03g51750D	/	/	7.85	7.33	AT5G62320	*MYB99*	MYB99 controls the exclusive production of tapetum di-glycosylated flavonols and hydroxycinnamic acid amides.	Battat et al., 2019
	BnaA06g13740D	/	/	/	3.08	AT1G19530	*RGAT1*	Direct target of RGA, plays an essential role in GA-mediated tapetum and pollen development.	Qian et al., 2021
	BnaA07g36770D	6.63	/	6.47	12.86	AT1G24520	*BCP1*	Male fertility gene acting on tapetum and microspore	Xu et al., 1995
	BnaA03g22590D	/	/	1.00	1.02	AT5G06100	*MYB33*	MYB domain protein 33	Jiang et al., 2021
	BnaA10g14090D	2.68	1.06	/	/	AT5G22260	*MS1*	RING/FYVE/PHD_zinc_finger_superfamily_protein	Lu et al., 2020
	BnaA03g22910D	4.29	5.87	7.51	6.68	AT1G02050	*LAP6*	Chalcone and stilbene synthase family protein	Lallemand et al., 2013
	BnaC03g26980D	3.58	6.53	7.72	6.95	AT4G00040		Chalcone and stilbene synthase family protein	Xu et al., 2010
	BnaA02g13290D	/	/	5.70	6.09	AT1G24400	*LHT2*	High-affinity transporter for neutral and acidic amino acids, expressed in tapetum tissue of anthers	Colcombet et al., 2005
Pollen mother cell and microspore division	BnaC01g42830D	6.64	6.75	4.81	7.01	AT3G47870	*SCP*, *ASL29, LBD27*	Required for normal cell division during pollen development. Mutant has extra cell in pollen of vegetative cell identity.	Oh et al., 2011
	BnaAnng17790D	9.39	8.87	6.11	7.92
	BnaA01g10390D	/	1.94	/	/	AT4G20050	*QRT3*	Encodes a polygalacturonase that plays a direct role in degrading the pollen mother cell wall during microspore development.	Preuss et al., 1994; Rhee and Somerville, 1998; Rhee et al., 2003; Ogawa et al., 2009
	BnaCnng18640D	/	1.84	/	/
	BnaA10g09760D	/	/	2.01	/	AT5G55590	*QRT1*	Encode a pectin methylesterase (PME) required for pectin degradation of the cell wall surrounding the pollen mother cell during pollen development.
	BnaC05g41210D	/	/	2.26	1.86	AT3G11980	*MS2*	Jojoba acyl CoA reductase-related male sterility protein,play specific roles during microspores released from tetrad.	Aarts et al., 1997
	BnaA05g27080D	/	/	1.86	1.57
Meiosis process	BnaA03g12670D	/	/	/	-1.17	AT5G52290	*SHOC1*	Encodes a protein with similarity to XPF endonucleases. Loss of function mutations have defects in meiosis.	Macaisne et al., 2008
	BnaA01g24420D	-1.29	-1.50	/	/	AT3G22880	*DMC1*	Expression of the AtDMC1 is restricted to pollen mother cells in anthers and to megaspore mother cells in ovules.	Muyt et al., 2009
	BnaCnng14320D	-1.12	-1.02	/	/
	BnaA08g25920D (MS5)	3.69	/	/	/		*MS5*	KAH0916352.1 Proteolysis; cysteine-type endopeptidase activity	Xin et al., 2016
	BnaA06g02920D	12.00	/	11.93	11.69	AT1G50240	*FU*	kinase_family_with_ARM_repeat_domain-containing_protein	Oh et al., 2005
Sporopollenin biosynthesis	BnaA03g22910D	4.29	5.87	7.52	6.68	AT4G00040		Chalcone and stilbene synthase family protein	Xu et al., 2010
	BnaC03g26980D	3.58	6.53	7.72	6.95
	BnaA10g19830D	/	/	2.84	1.96	AT1G68540	*TKPR2, CCRL6*	NAD(P)-binding Rossmann-fold superfamily protein	Wang et al., 2018
	BnaA07g27190D	/	-3.11	/	/
	BnaC06g30130D	/	/	5.47	5.26
Pollen exine formation	BnaC05g28280D	/	/	2.15	1.66	At1g33430	*UPEX1, KNS4*	UPEX1 is arabinogalactan b-(1,3)-galactosyltransferase involved in the formation of pollen exine.	Dobritsa et al., 2011
	BnaCnng54730D	/	/	1.92	1.40
	BnaA03g14100D	/	/	/	6.60	At2g30710		Ypt/Rab-GAP domain of gyp1p superfamily protein	Dobritsa et al., 2011
	BnaC03g17060D	/	/	/	6.52
	BnaC04g29090D	/	/	2.49	/	At4g14080	*MEE48*	O-Glycosyl hydrolases family 17 protein.	
	BnaC07g22030D	/	/	/	1.31	AT2G02970	*APY6*	Encodes a putative apyrase involved in pollen exine pattern formation and anther dehiscence.	Yang et al., 2013
	BnaA06g33910D	/	/	/	1.61
	BnaC09g43680D	/	/	2.72	3.19	AT5G13390	*NEF1*	Required for normal pollen development and lipid accumulation within the tapetum.	Ariizumi et al., 2010
	BnaA06g29150D	7.03	3.50	/	/	AT5G28470	*NPF2.8, FST1*	The Tapetal Major Facilitator NPF2.8 is Required for Accumulation of Flavonol Glycosides on the Pollen Surface	Grunewald et al., 2020
	BnaC03g26220D	/	1.31	1.95	2.51	AT2G29940	*ABCG31*	ABCG9 and ABCG31 participate in Pollen Fitness and the Deposition of Steryl Glycosides on the Pollen Coat	Choi et al., 2014
	BnaCnng55560D	2.64	7.09	8.26	12.07	AT1G75930	*EXL6*	Extracellular_lipase_6	Xu et al., 2014
	BnaC02g23340D	2.05	7.17	10.15	12.54
	BnaA02g17590D	2.94	7.83	8.06	11.18
Pollen maturation	BnaCnng42600D	/	/	/	6.51	AT5G15100	*PIN8*	Encodes an auxin transporter with a strong expression in a male gametophyte. Mutant studies reveal a role for auxin transport in regulating pollen development and function.	Bosco et al., 2012
	BnaA10g17940D	/	/	-1.30	/	AT5G16530	*PIN5*	Encodes PIN5, an atypical member of the PIN family. It acts together with PIN8 in affecting pollen development and auxin homeostasis.	Ding et al., 2012
	BnaC08g34690D	10.07	8.80	10.57	11.20	AT2G22950	*ACA7*	Encodes a putative auto-regulated Ca2+-ATPase located in the plasma membrane involved in transporting Ca2+ outside developing pollen grains. This activity is important to support normal pollen development, particularly the progression to uninucleated microspores to bicellular pollen grains.	Lucca and Leon, 2012
	BnaA02g13560D	/	/	6.25	6.34	AT1G68090	*ANN5*	Encodes a calcium-binding protein annexin (AnnAt5). Plays a vital role in pollen development *via* Ca2+ dependent membrane trafficking.	Zhu et al., 2014
	BnaC02g45910D	6.45	/	4.55	4.89
	BnaC06g42760D	/	5.73	6.20	5.58	AT3G60460	*DUO1*	Encodes an R2R3 myb transcription factor that is required for male gamete formation, specifically for entry of the generative cell into mitosis.	Rotman et al., 2005
	BnaA07g18670D	/	/	6.38	6.00
	BnaC08g30650D	6.03	/	5.16	/
	BnaA09g38510D	/	/	6.14	/
	BnaA07g33840D	8.72	7.06	8.03	8.03	AT1G77980	*AGL66*	Encodes a member of the MIKC family of transcriptional regulators. AGL66 is expressed in pollen. Involved in late stages of pollen development and pollen tube growth.	Adamczyk and Fernandez, 2009; Xia et al., 2014
	BnaC06g38460D	9.57	7.28	9.69	10.89
	BnaC08g05830D	9.15	7.59	7.52	8.28	AT1G22130	*AGL104*	Encodes a member of the MIKC family of transcriptional regulators. AGL104 is expressed in pollen. Involved in late stages of pollen development and pollen tube growth.	Adamczyk and Fernandez, 2009
	BnaC07g14030D	8.26	4.95	4.11	7.16
	BnaA07g10680D	7.16	/	6.91	7.95
	BnaA08g20930D	8.63	7.41	8.48	8.87
	BnaCnng07450D	4.47	4.64	2.24	3.00	AT3G57390	*AGL18*	Encodes a MADS-box containing protein likely to be a transcription factor that is expressed in endosperm and developing gametophytes.	Verelst et al., 2007
Stamen development	BnaA03g27440D	-1.50	/	-2.15	/	AT3G01530	*MYB57*	Member of the R2R3 factor gene family.MYB57 interacts with JAZ proteins, and functions redundantly with MYB21 and MYB24 to regulate stamen development.	Cheng et al., 2009
	BnaA04g13670D	/	/	/	-1.29	AT2G23470	*RUS4*	DUF647 domain containing protein. Mutants are male sterile with defects in endothecium, tapetum and stamen maturation.	Chen et al., 2020

In previous studies, *MS5* (BnaA08g25920D) was a key gene that caused sterility in *B. napus*. It participated in meiosis progression by regulating chromosome configuration during early prophase I (Xin et al., 2016). *DMC1* and *FU* played important roles during meiosis process in *Arabidopsis* (Oh et al., 2005; Muyt et al., 2009). In our transcriptome data, *MS5*, *DMC1*, and Fu were differentially expressed in DGMS (**Table 2**). And the abnormal pollen development in our sterile lines of DGMS arose from the meiosis process. Therefore, *MS5*, *DMC1*, and Fu may be key genes involved in the fertility regulation of DGMS. *MS3* and *Rf* were key genes controlling fertility in RGMS of *B. napus*, and the abnormal vacuolization of the tapetum during the tetrad stage was associated with male sterility (Wan et al., 2010; Deng et al., 2016). We found the expression level of *MS3* (BnaC09g41260D) decreased in both 6251A and 6284A. And *Rf* was also differentially expressed in 6251AB and 6284AB. Coincidentally, abnormal vacuolization of the tapetum was also found in 6251A. So *MS3* and Rf were probably key genes in RGMS. In addition, many other genes involved in tapetum development were specifically differentially expressed in RGMS. These genes included *AMS* (BnaC07g05950D, BnaA07g03340D), *MYB99* (BnaC03g51750D), *MYB33* (BnaA03g22590D), and *LHT2* (BnaA02g13290D). Therefore, *MS3* (BnaC09g41260D), *Rf*, *AMS*, *MYB99*, *MYB33*, and *LHT2* may be key genes involved in the fertility regulation of RGMS.

### Validation of differentially expressed genes by qRT-PCR

**Figure 11:**
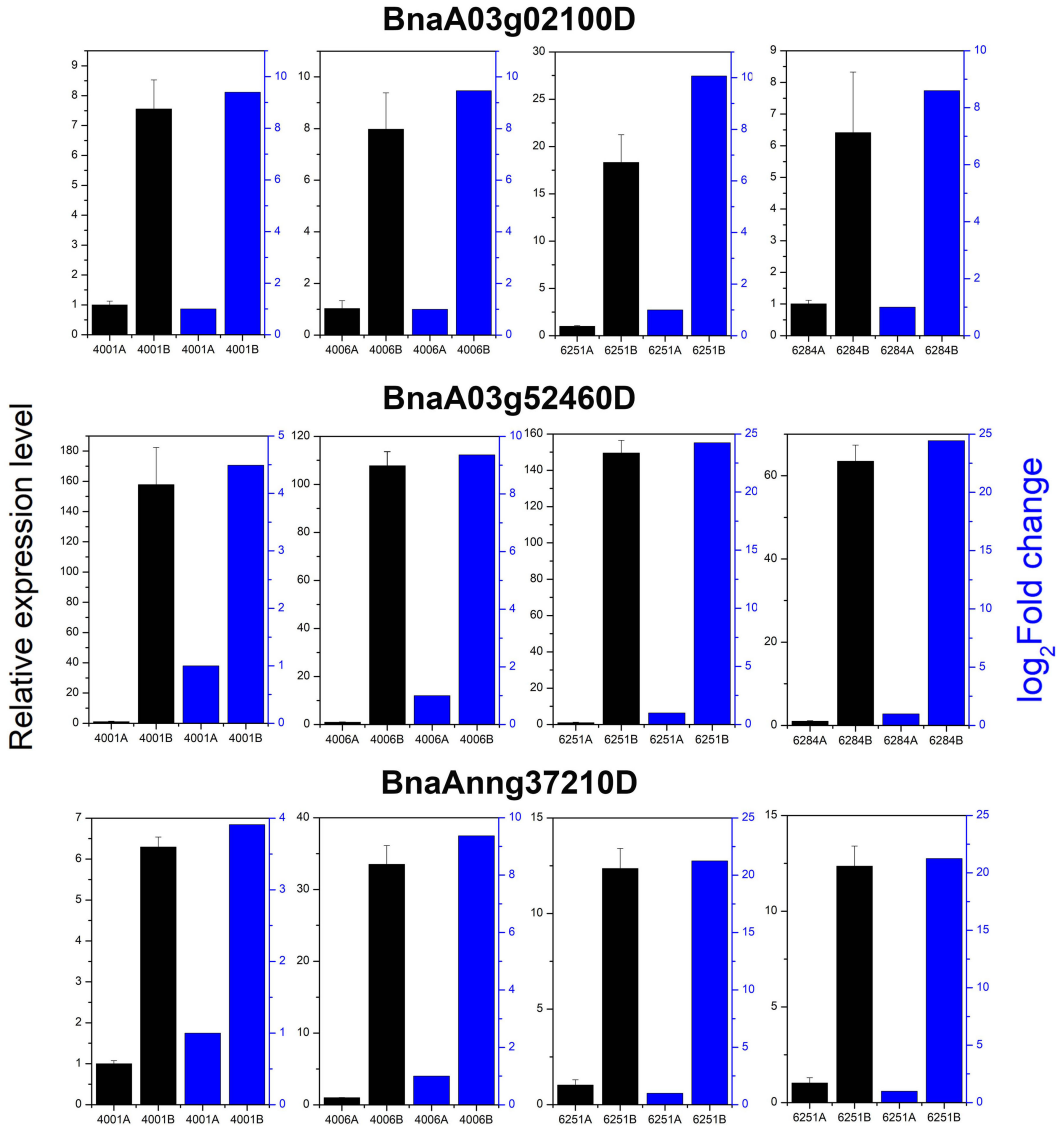
Relative expression analysis of three DEGs by transcriptome and qRT-PCR analysis. All qRT-PCR reactions were prepared in triplicate for each sample. The blue columns indicate the gene relative expression level from transcriptome sequencing. The black columns indicate the gene relative expression level obtained from qRT-PCR analysis.

qRT-PCR was conducted to verify the expression levels of three randomly selected DEGs, namely, BnaA03g02100D, BnaA03g52460D, and BnaAnng37210D in 4001AB, 4006AB, 6251AB, and 6284AB, respectively. These three genes were upregulated in the fertile lines, which largely agreed with the expression trend in transcriptome results (**Figure 11**). The qRT-PCR results validated the accuracy and reliability of the obtained transcriptome data.

## Discussion

Rapeseed is one of the primary sources of vegetable oil for human nutrition in the world, which occupies an important position in China’s edible oil supply. In recent years, transcriptome sequencing has been widely used for the identification of candidate genes related to various agronomic traits of rapeseed, which greatly facilitate the identification of functional genes underlying important yield and quality traits and offer abundant resources for breeding excellent rapeseed varieties. By using transcriptome sequencing, developing pods of German cultivar Sollux and Chinese inbred line Gaoyou were studied to explore the molecular mechanism of oil-related biological processes. A total of 33 genes such as Sac domain-containing phosphoinositide phosphatase (*Sac-PIP*) were identified as candidate genes that affect seed oil content. *Sac-PIP* is a lipid-related gene, which participates in phospholipid signaling pathway (Xu et al., 2015). By transcriptome and metabolome correlation analysis, Tan et al. revealed the regulatory network for lipid synthesis in developing *B. napus* embryos (Tan et al., 2019). Transcriptome analysis was also widely used to identify the abiotic stress-related genes. By using transcriptomics, the mechanism of seed response to heat stress at 40 or 60°C was explored. The results show that 442 DEGs were identified in seeds after heat stress (Gao et al., 2021). By using transcriptome and proteome analyses, the molecular mechanisms underlying changes in oil storage under drought stress in *B. napus* L. were revealed. Lots of major genes involved in ABA signal transduction, such as *BnaA06g40220D*, *BnaA03g13020D*, *BnaA01g23120D*, and *BnaA05g08020D*, were highly expressed under drought stress (Li et al., 2021). Long et al. used transcriptome analysis to explore salt-stress-responding genes by using digital gene expression (DGE) at 0, 3, 12 and 24 h after H_2_O (control) and NaCl treatments on *B. napus* roots at the germination stage. A total of 163 genes were differentially expressed at all the time points. These genes were new candidate salt-stress-responding genes, which may function in novel putative nodes in the molecular pathways of salt stress resistance (Long et al., 2015). Li et al. performed a global dynamic transcriptome programming of rapeseed anther at different development stages. The transcriptome sequencing results revealed that 35,470 transcripts were expressed in at least one of the anther development stages from the pollen mother cell stage to the mature pollen stage (Li et al., 2016). Therefore, much more genes that are involved in pollen development need to be discovered.

In our study, the potential genes related to pollen development and the formation of genetic male sterility were identified by constructing and sequencing 24 transcriptome libraries for the flower buds from the fertile and sterile lines of two RGMS and two DGMS lines. A total of 23,554 redundant DEGs with over two-fold change between sterile and fertile lines were obtained, including 4,820 DEGs (4,251 upregulated and 569 downregulated) in 4001AB, 4,257 DEGs (3,857 upregulated and 400 downregulated) in 4006AB, 6,093 DEGs (5,230 upregulated and 863 downregulated) in 6251AB, and 8,384 DEGs (7,347 upregulated and 1,037 downregulated) in 6284AB. The number of DEGs identified in our study was much more than those in previous studies. Our findings provide a global view of genes that are potentially involved in GMS occurrence. In the four GMS lines, the number of upregulated genes was much greater than the number of downregulated genes. A similar phenomenon was also observed in previous reports (Wu et al., 2007). Maybe this phenomenon in our study was caused by the following reasons. The fertile and sterile plants of the homozygous GMS two-type line have similar genetic background (Li et al., 1988). Consequently, the fertile and sterile plants should have a similar gene expression pattern before the key stage (tetrad stage) for fertility control. Thereafter, the male gametes of the sterile lines began to stop its development at the meiotic stage or pre-tetrad stage, and its tapetum and pollen mother cell also began to degrade to form empty pollen sacs. As a result, a large number of pollen development-related genes had low or undetectable expression in the sterile lines, while most of them were normally expressed in fertile lines. Therefore, the number of up-regulated genes in fertile lines was significantly greater than the down-regulated genes. To verify the expression of genes in transcriptome sequencing, three genes were randomly selected, and their expression levels were analyzed by qRT-PCR. The results of qRT-PCR largely agreed with the transcriptome sequencing results.

In our sequencing results, 2,214 upregulated DEGs and 52 downregulated DEGs were shared between 4001AB and 4006AB. And 4,196 upregulated DEGs and 283 downregulated DEGs were shared between 6251AB and 6284AB. In total, 1,545 DEGs were shared between the two DGMS and RGMS lines. We found the DEGs shared between two DGMS lines or two RGMS lines were not too much. In theory, the same dominant sterility genes cause sterility of 4001A and 4006A. And the same recessive sterility genes cause sterility of 6251A and 6284A. However, the genetic backgrounds of 4001AB and 4006AB, or the genetic backgrounds of 6251AB and 6284AB, are very different. In addition, their flowering period and agronomic characters are also quite different. Perhaps these factors lead to the small number of DEGs shared between two fertile and sterile lines.

In previous studies, many Arabidopsis genes have been reported to be involved in anther and microspore development processes. To investigate the potential DEGs related to pollen development processes, 13,031 Arabidopsis genes related to anther, pollen, and microspore development were downloaded from TAIR website. And 6,099 homologous DEGs in *B. napus* were identified, which might be involved in anther, pollen and microspore development. We further screened 47 Arabidopsis genes that have been reported in previous studies to be involved in anther and microspore developmental processes, such as *AGL18* (Verelst et al., 2007), *AGL66* (Adamczyk and Fernandez, 2009; Xia et al., 2014), *AGL104* (Adamczyk and Fernandez, 2009), *SCP* (Oh et al., 2011), *ACA7* (Lucca and Leon, 2012), *MS2* (Aarts et al., 1997), and *QRT3* (Rhee et al., 2003). And 74 DEGs were identified to be their homologous genes, containing *MS2*, *QRT1*, and *QRT3*, etc. It is worth noting that the *MS5* (BnaA08g25920D) (Xin et al., 2016), *DMC1*, *FU* play specific roles in meiosis process (Oh et al., 2005; Muyt et al., 2009). And *MS3* (BnaC09g41260D), *Rf, AMS, MYB99, MYB33*, and *LHT2* play important roles in tapetum development (Colcombet et al., 2005; Xu et al., 2010; Dong et al., 2012; Li et al., 2012; Deng et al., 2016; Battat et al., 2019; Jiang et al., 2021). Coincidentally, we found the abnormal pollen development in our sterile lines of DGMS arose from the meiosis process, and the abnormal pollen development in RGMS occurred before tetrad stage. Therefore, we speculated that the abnormal meiosis or tapetum development might be an important cause of sterility. And *MS5* (BnaA08g25920D), *DMC1*, and *Fu* may be key genes involved in the fertility regulation of DGMS. *MS3* (BnaC09g41260D), *Rf* (BnaA01g00190D), *AMS*, *MYB99*, *MYB33*, and *LHT2* may be key genes involved in the fertility regulation of RGMS.

*MYB* in rice (*OsGAMYB*) and *Arabidopsis* (*AtMYB33* and *AtMYB65*) were identified as plant GMS genes. Loss-of-function mutations of *OsGAMYB* failed to produce normal pollen grains and resulted in a sterile panicle of rice (Tsuji et al., 2006). *B. napus MIR159* overexpression in *A. thaliana* resulted in reduced transcripts of *AtMYB33* and *AtMYB65* and decreased seed setting rate (Jiang et al., 2021). *AtMYB101* participates in pollen tube reception possibly by controlling the expression of downstream genes (Liang et al., 2013). In the present study, the transcripts of *MYB33*, *MYB65*, and *MYB101* were analyzed between fertile and sterile lines. More transcripts of *MYB33* and *MYB101* accumulated in the fertile lines (**Figure S3D**). Other TFs such as NAC and WRKY family genes may play critical roles in pollen development and plant fertility (Wang et al., 2019; Liu et al., 2020).

## Conclusions

This study revealed that the abnormal pollen development in DGMS lines might start at the meiotic stage, and abnormal pollen development in RGMS lines probably occurred before the tetrad stage. In total, 24 transcriptome libraries were constructed and sequenced for the flower buds from the fertile and sterile lines of RGMS and DGMS. Based on the results, a large number of DGEs was obtained. Some DEGs were candidate genes for rapeseed breeding, and they are expected to provide new insight into the molecular mechanisms underlying GMS.

## Data availability statement

The datasets presented in this study can be found in online repositories. The name of the repository and accession number can be found below: SRA, NCBI; PRJNA879090.

## Author contributions

JJ and LY conceived the project and research plan. JJ drafted the manuscript. JJ and PX analyzed the sequencing data, performed the experiments, and finished the manuscript. JYZ, XZ, and JFZ planted and observed the DGMS plants. YL, MJ, and WW planted and observed the RGMS plants. All authors contributed to the article and approved the submitted version.

## Funding

This work was supported by grants from the National Key Research and Development Program of China (Grant No.2018YFD0100602), National Modern Agricultural Industry Technology System (Grant No. CARS-12), and Shanghai Agriculture Applied Technology Development Program, China (Grant No.T20210219).

## Conflict of interest

The authors declare that the research was conducted in the absence of any commercial or financial relationships that could be construed as a potential conflict of interest.

## Publisher’s note

All claims expressed in this article are solely those of the authors and do not necessarily represent those of their affiliated organizations, or those of the publisher, the editors and the reviewers. Any product that may be evaluated in this article, or claim that may be made by its manufacturer, is not guaranteed or endorsed by the publisher.

## Supplementary material

Supplementary Figure 1 Pearson correlation and principal component analysis. **(A)** Representative correlation plots between A and B lines. Plots display between log_10_(FPKM), normalized read counts. **(B)** PCA of the transcriptome expression profiler among samples. Genotype is represented by different color. The relationships are calculated by FPKM values.

Supplementary Figure 2 Analysis of the expression levels of DEGs. **(A)** The downregulated genes in A lines of GMS, numbers in each box are FPKM values. Genes specifically involved in DGMS (**B** and **C**) and RGMS (**D** and **E**). The expression levels are calculated by FPKM values. Significant differences were calculated by performing paired t-test between the FPKM values of A and B lines. ***P* < 0.01, “ns” means no significant difference.

Supplementary Figure 3 Shared DEGs possibly involved in GMS. **(A–C)** *BnaAnng17790D*, *BnaC08g05830D*, and *BnaA07g33840D* were selected, and their expression levels were calculated using FPKM values. Significant differences were calculated by performing a paired t-test between FPKM values of A and B lines. ***P* < 0.01. **(D)** Analysis of the expression levels of MYB TFs. Numbers in each box are FPKM values.

## Abbreviations

GMS, Genic male sterility; RGMS, recessive genic male sterility; DGMS, dominant genic male sterility; CMS, cytoplasmic male sterility; GO, Gene ontology; KEGG, Kyoto Encyclopedia of Genes and Genomes; DEGs, Differentially expressed genes; qRT-PCR, Quantitative real-time polymerase chain reaction.
