# Supplementary figures and images for: Global transcriptome analysis reveals potential genes associated with genic male sterility of rapeseed (Brassica napus L.)
Source: Front Plant Sci. 2022 Oct 21;13:1004781. doi: 10.3389/fpls.2022.1004781 (PMC9635397; doi:10.3389/fpls.2022.1004781)

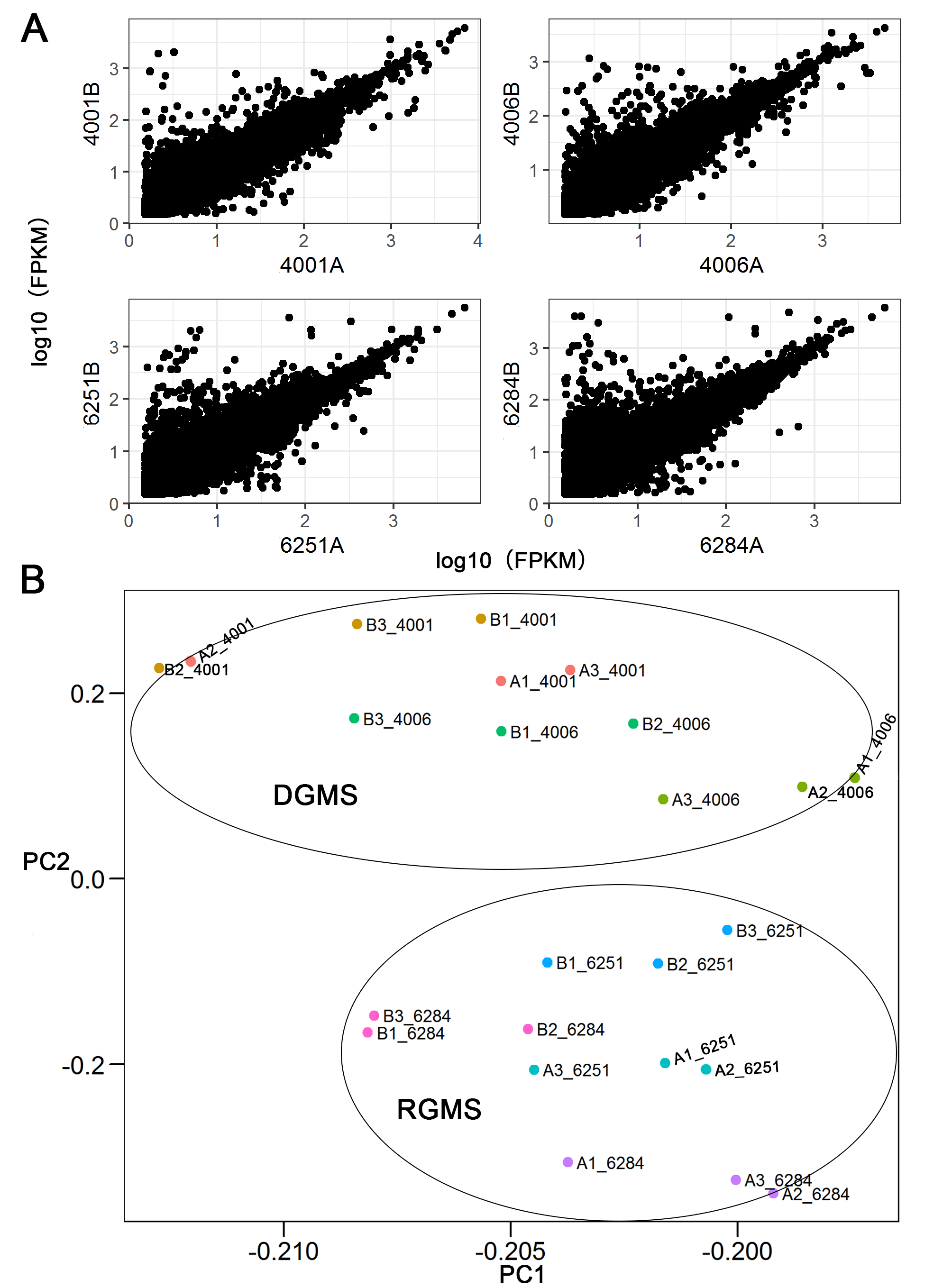

Supplement: Supplementary Figure 1 — Pearson correlation and principal component analysis. (A) Representative correlation plots between A and B lines. Plots display between log10(FPKM), normalized read counts. (B) PCA of the transcriptome expression profiler among samples. Genotype is represented by different color. The relationships are calculated by FPKM values. [file Image_1.tif]

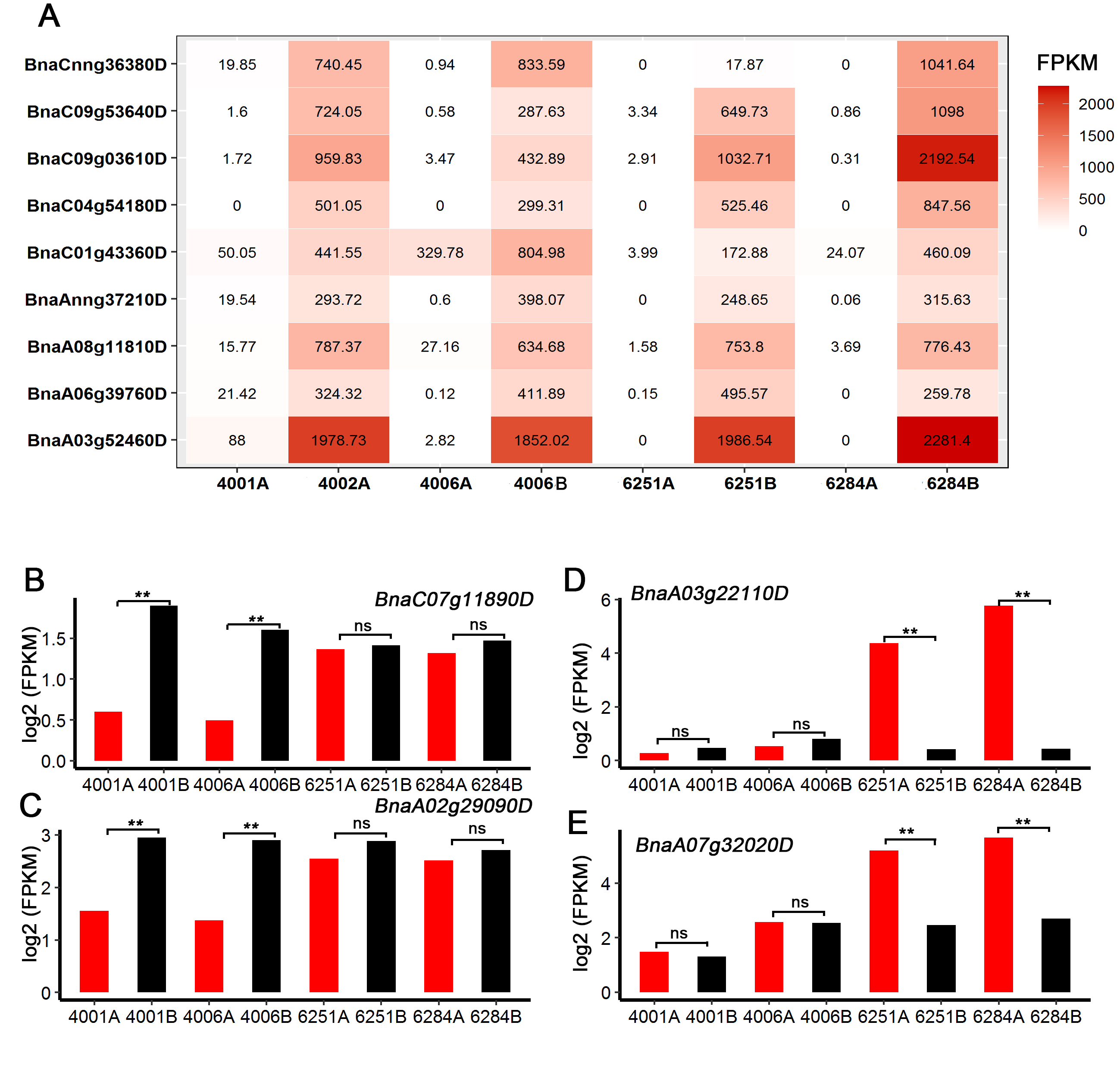

Supplement: Supplementary Figure 2 — Analysis of the expression levels of DEGs. (A) The downregulated genes in A lines of GMS, numbers in each box are FPKM values. Genes specifically involved in DGMS (B and C) and RGMS (D and E). The expression levels are calculated by FPKM values. Significant differences were calculated by performing paired t-test between the FPKM values of A and B lines. **P < 0.01, “ns” means no significant difference. [file Image_2.tif]

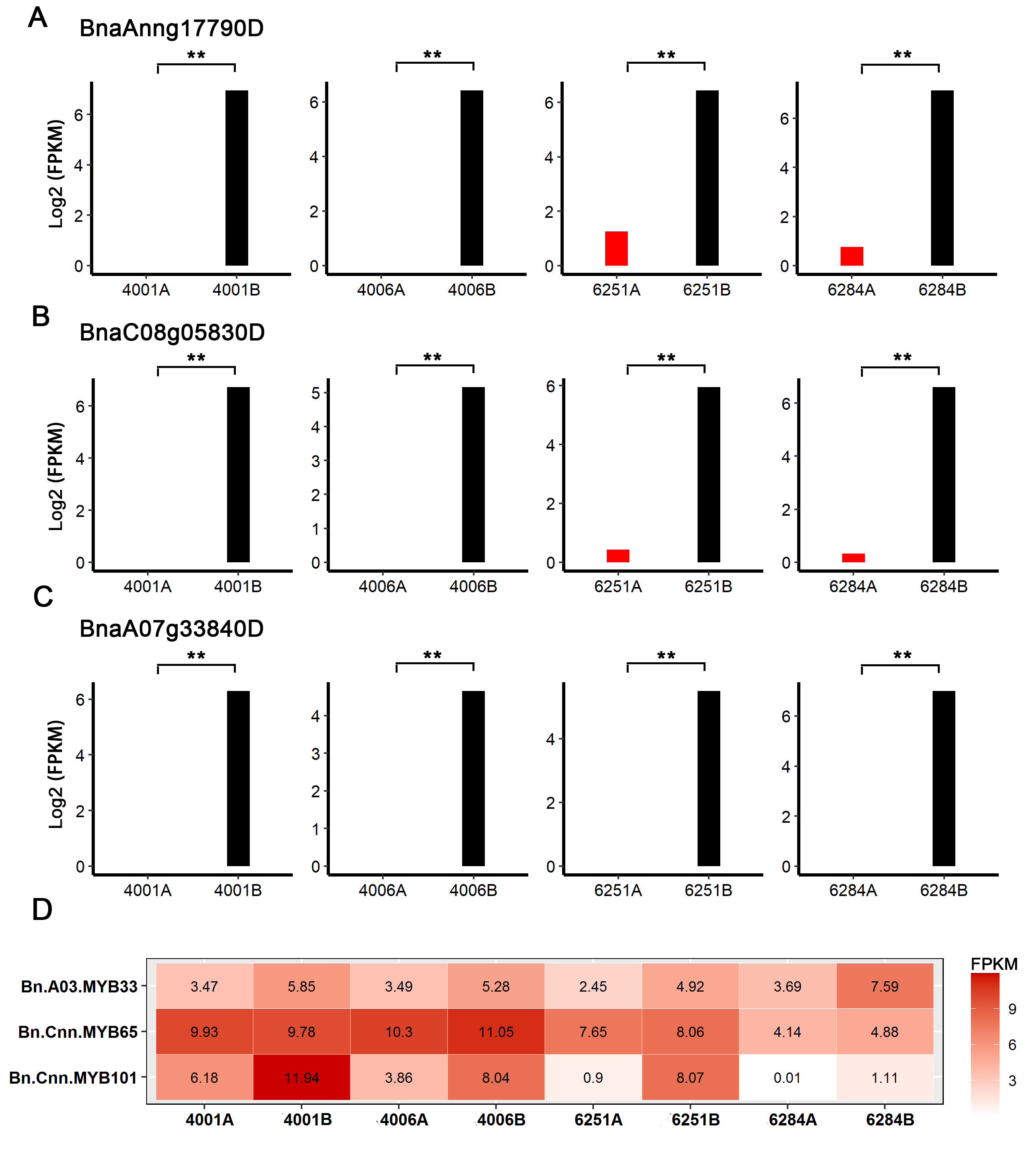

Supplement: Supplementary Figure 3 — Shared DEGs possibly involved in GMS. (A–C) BnaAnng17790D, BnaC08g05830D, and BnaA07g33840D were selected, and their expression levels were calculated using FPKM values. Significant differences were calculated by performing a paired t-test between FPKM values of A and B lines. **P < 0.01. (D) Analysis of the expression levels of MYB TFs. Numbers in each box are FPKM values. [file Image_3.tif]
